# Supplementary material for: Cloud BioLinux: pre-configured and on-demand bioinformatics computing for the genomics community
Source: BMC Bioinformatics. 2012 Mar 19;13:42. doi: 10.1186/1471-2105-13-42 (PMC3372431; doi:10.1186/1471-2105-13-42)
Supplement: Additional file 1 — Supplementary 1 Cloud BioLinux software documentation in the form of a mini, self-contained website. Users need to download and uncompress the .zip file, and open through a web browser the "index.html" file available on the main directory. (ZIP 1823 kb). [file 1471-2105-13-42-S1.ZIP › Cloud-BioLinux-Package-Documentation/docs/squint.html]

Bio-Linux Software Documentation Pages

Back to search form

## squint

|  |  |
| --- | --- |
| Name | squint |
| Description | **Squint** is a multiple alignment program and editor.  Squint allows automated, progressive alignments as well as manual editing. Nucleotide or amino acid files can be imported in a number of formats. Alignments can be viewed in two windows, where each window can have a different translation of the alignment. Squint allows the user to do partial progressive alignments and also to align codon sequences.  Squint can be started up by typing `squint` on the command line or by choosing squint from the Bioinformatics drop down menu under the Applications menu on your Bio-Linux desktop.  Help can be found under the Help menu when you are running squint. |
| Homepage | http://www.cebl.auckland.ac.nz/software1.php |
| Remote Documentation | http://bioinformatics.oxfordjournals.org/cgi/content/full/23/12/1553 |
